# Vitamin B12 concentrations in pregnant Colombian women: analysis of nationwide data 2010

**DOI:** 10.1186/s12884-016-0820-4

**Published:** 2016-02-01

**Authors:** Robinson Ramírez-Vélez, Jorge Enrique Correa-Bautista, Javier Martínez-Torres, José Francisco Meneses-Echávez, Felipe Lobelo

**Affiliations:** Centro de Estudios para la Medición de la Actividad Física «CEMA». Escuela de Medicina y Ciencias de la Salud, Universidad del Rosario, Bogotá, D.C Colombia; Grupo GICAEDS. Programa de Cultura Física, Deporte y Recreación, Universidad Santo Tomás, Bogotá, D.C Colombia; Norwegian Knowledge Centre for the Health Services, Oslo, Norway; Hubert Department of Global Health, Rollins School of Public Health, Emory University, Atlanta, GA USA

**Keywords:** Nutrition, Pregnancy, Vitamin B12, Deficiency, Prevalence

## Abstract

**Background:**

Vitamin B12 deficiency is associated with many adverse health outcomes and is highly prevalent worldwide. The present study assesses the prevalence and socio-demographic factors associated with vitamin B12 deficiency in a representative sample of pregnant women in Colombia.

**Method:**

We used data from the cross-sectional, nationally representative survey (ENSIN, 2010). A total of 1.781, (13–49 years old) pregnant women were enrolled. Serum Vitamin B12 a concentration was determined by chemiluminescence and sociodemographic date was assessed by computer-assisted personal interview technology. Multivariate analyses using unordered multinomial logistic regression models were conducted in the main analysis.

**Results:**

Vitamin B12 concentrations ranged from 45 to 1000 pg/mL (mean 299.2 pg/mL, 95 % CI 290.6 to 303.7 pg/mL). A total of 18.6 % of pregnant women had vitamin B12 concentrations below 200 pg/mL and 41.3 % had concentrations between 200 and 300 pg/mL. Being of indigenous ethnicity, living in the east and living in a rural area showed the lowest mean values (273.2 pg/mL, 270.8 pg/mL and 290.1 pg/mL, respectively). The multivariate logistic regression shows that pregnant women belonging to the indigenous ethnic group OR 2.2, (95 % CI 1.1 to 4.3), living in the pacific region (west) OR 4.4, (95 % CI 2.8 to 6.9), or national territories (south) OR 2.3, (95 % CI 1.4 to 3.7) were associated with a higher probability of serum vitamin B12 deficiency.

**Conclusion:**

The prevalence of vitamin B12 deficiency in Colombian pregnant women is substantial. Factors associated with depletion among pregnant women should be considered for future interventions in countries experiencing nutritional transition.

## Background

Maternal nutritional status is a determining factor of fetal growth and birth weight [1]. Globally, the prevalence of maternal malnutrition ranges from 10 % to 20 %; however, it is more common among low-income populations [2]. Short-term changes in maternal nutrition can result in fetal growth retardation and complications unique to human pregnancy, such as preeclampsia, eclampsia and pregnancy-induced hypertension [3]. The long-term effects of growth retardation have been associated with an increased risk of non-communicable diseases, such as hypertension, coronary heart disease, insulin resistance, type 2 diabetes mellitus and hyperlipidemia [4, 5].

Given the risk of malnutrition in developing countries, it is necessary to measure its prevalence in vulnerable populations, such as children, pregnant women and minorities, to identify high risk groups and develop preventive interventions [6]. To estimate the magnitude of this problem, the use of direct indicators to assess various nutrient levels, such as iron, vitamin A, zinc and vitamin B12, has been proposed. Lack of these nutrients in the diet also contributes to Vitamin B12 deficiency. These biochemical indicators determine the concentrations of micronutrients based on factors such as absorption, which reflects the nutritional status of biological processes, including cognitive development, physical growth and immune response [7]. Vitamin B12 deficiency and folate deficiency causes neural tube defects, such as spina bifida and anencephaly, due to the failure of cell growth and replication in the fetus and placenta [8]. Therefore, children who are breastfed by vegetarian mothers are at greater risk of serious complications related to vitamin B12 deficiency, which include hematologic [megaloblastic anemia], neurologic [hypotonia, ataxia, developmental delays] and gastrointestinal [inflammatory complications] symptoms [3, 4]. The consequences of anemia are not limited to poor pregnancy outcomes, impaired physical and cognitive development, and increased risk of morbidity in children but also affect national productivity and economics [3].

Vitamin B12 deficiency is recognized as an important health problem and different studies involving different age groups and both genders have underscored the magnitude of vitamin B12 deficiency as a major public health challenge for improving the health status especially in refugee camps and among vulnerable at-risk groups. Currently, there are few global reports on the prevalence of vitamin B12 deficiency in pregnant women, in particular for low-to-middle income countries (LMIC´s) experiencing rapid nutrition transitions such as Latin America or Africa. LMIC´s included Colombia are environment to assess malnutrition in pregnant women because the prevalence of both underweight and overweight individuals is relatively high; furthermore, an obesity gradient that includes women from even the poorest households has been reported [2, 9]. However, to the best of our knowledge, associations of Vitamin B12 deficiency with various socio-demographic factors that could help identify risk groups and offer information to better design interventions has not been investigated in a nationally representative sample in the Americas.

Therefore, this study aimed to evaluate the prevalence and sociodemographic factors associated with vitamin B12 deficiency in a representative sample of pregnant women in Colombia.

## Methods

### Design

The Colombian National Nutrition Survey (ENSIN) was carried out together with the National Demographic and Health Survey by Asociación Pro-bienestar de la Familia Colombiana (PROFAMILIA), a nonprofit organization focusing on reproductive health [10]. Details of the survey have been published elsewhere [10]. In brief, participants were selected to represent 99 % of the country’s population using a multistage stratified sampling scheme. Subsamples were randomly drawn to estimate departmental, subregional, regional, and/or national-level estimates of specific nutrition problems among individuals 0–64 y. All municipalities from the thirty-two departments in the country were grouped into strata based on similar geographic and sociodemographic characteristics. The survey included 50,670 households, representing 4,987 clusters from 258 strata. The first author applied to the PROFAMILIA-ENSIN and obtained permission to use the publicly available data for research and teaching learning purpose. Further details can be obtained from the website of PROFAMILIA-ENSIN (http://www.icbf.gov.co/portal/page/portal/PortalICBF/Bienestar/ENSIN1).

### Participants

Of the 12,437 women of childbearing age (13–49 years), 1,781 (14.3 %) were pregnant (mean age 24.4 years). The study was conducted according to the guidelines laid down in the Declaration of Helsinki. All participants provided written informed consent and the Research Ethics Review Board at the Colombian Institute of Family Welfare approved the survey protocol. A comprehensive verbal description of the nature and purpose of the study and its experimental risks was given to all participants and, for participants under 18, their parents/guardians. All participants and parents/legal guardians of participants under 18 provided written informed consent before entering the study. The Ethical Committee of the PROFAMILIA provided ethical approval prior to data collection. To conduct the present analysis using the ENSIN 2010 database, the Manuela Beltrán University Research Ethics exempted the project [Resolución 8430 de 1993; Ministerio de Salud de Colombia].

### Data sources

Serum vitamin B12 was quantified in these samples using the method of direct chemiluminescence (ADVIA Centaur equipment, Siemens). This method offers high sensitivity and is less costly, easier to implement and safer than microbiological, chromatographic or spectrophotometric assays [7]. The main outcomes of interest were mean serum vitamin B12 concentrations (pg/mL) and the prevalence of vitamin B12 deficiency (serum concentration <200 pg/mL) and marginal deficiency (serum concentration 200 to 300 pg/mL) [11]. All participants in this study had non-fasting blood samples.

The following sociodemographic variables were defined as associated factors: age (13 to 17, 18 to 29 and 30 to 49 years old); urbanicity (urban *or* rural); ethnicity grouped as: a) indigenous (indigenous ethnicity is considered main minority groups: approx. 1,378.884 (3,4 %) people belonging to various indigenous groups (i.e.: Emberá, Misak, Ika, Kankuamo, Nasa, Wayuu, Awuá and Mokane) [7], b) black or afro-colombian and c) others (mestizo); geographic region: a) Atlantic (North), b) Eastern, c) Central, d) Pacific (West), e) Bogota and f) National Territories (South region, included seven department: Arauca, Casanare, Putumayo, Amazonas, Guainía, Vaupés y Vichada); and social or socioeconomic status determined by the System of Identifying Potential Beneficiaries of Social Programs (SISBEN for its *Spanish initials*). The SISBEN is a system designed by the Colombian National Government to identify families who could benefit from social programs. It takes into account sociodemographic characteristics (family composition, employment status, family income, and educational level), living conditions (construction type and materials), and access to public utilities (sewer, electricity, potable water, and garbage collection). Households with SISBEN levels I or II are the most vulnerable and targeted in social programs. Here, SISBEN level IV includes levels IV–VI and is considered the least vulnerable sector of society. For this study we classified SISBEN scores into 4 categories (I, II, III and IV or more).

### Data analysis

First, we conducted an exploratory analysis for quantitative variables (vitamin B12 concentrations) and relative frequencies (sociodemographic variables) using the *Pearson χ*^2^ test with and without the *Yates* correction. To estimate the relationship between vitamin B12 deficiency and sociodemographic variables in pregnant women (age, urbanicity geographic region, ethnicity and socioeconomic level-SISBEN), binary logistic regression models were used. The first adjusted model was for age, the second model was based on ethnic group, geographic area, socioeconomic levels and urbanicity, and the third model was adjusted by age, ethnic group, geographic area, socioeconomic levels and urbanicity. Odds ratios were considered a confounder if they shifted the model in a constant direction with a proportional increase in the exposure level of at least 10 %. All analyses were conducted with the use of the complex survey design routines of the SPSS Statistical software package version 20 (SPSS, Chicago, IL, USA).

## Results

Vitamin B12 concentrations ranged from 45 to 1000 pg/mL (mean 299.2 pg/mL, 95 % CI 290.6 to 303.7 pg/mL). Being of indigenous ethnicity, living in the east and living in a rural area showed the lowest mean values (273.2 pg/mL, 270.8 pg/mL and 290.1 pg/mL, respectively). A total of 18.6 % of pregnant women had vitamin B12 concentrations below 200 pg/mL and 41.3 % had concentrations between 200 and 300 pg/mL. Indigenous women who lived in National Territories (South) or had a higher socioeconomic level (Scores 4 or more SISBEN) showed a greater vitamin B12 deficiency (30.8 %, 22.3 % and 20.2 %, respectively) (Table 1).

**Table 1:** Prevalence and socio-demographic factors associated with vitamin B12 deficiency and marginal vitamin B12 deficiency in a representative sample of pregnant women in Colombia

Characteristics	Adequate B12 concentration		Marginal vitamin B12 deficiency		Vitamin B12 deficiency	
	n	%^a^, 95 % CI^b^	n	%^a^, 95 % CI^b^	n	%^a^, 95 % CI^b^
Total	697	40.1 (38.1–41.8)	737	41.3 (39.4–43.0)	347	18.6 (16.8–20.3)
Age (years)						
13 to 17	101	42.2 (37.2–45.7)	94	39.4 (33.9–42.2)	50	18.4 (14.1–21.5)
18 to 29	435	39.6 (37.3–41.6)	489	42.3 (40.2–44.2)	218	18.1 (16.0–20.0)
30 to 49	161	40.3 (36.6–43.0)	154	39.5 (35.9–42.4)	79	20.2 (16.1–23.4)
Socioeconomic level						
Level I	417	41.1 (38.7–43.1)	418	40.8 (38.4–42.9)	200	18.1 (15.8–20.0)
Level II^c^	62	34.4 (28.3–38.5)	78	49.9 (44.6–53.5)	30	15.7 (10.5–19.3)
Level III^c^	48	48.6 (41.1–53.3)	51	33.0 (27.5–36.6)	25	18.4 (11.4–22.9)
Level IV or more	170	38.4 (35.1–41.0)	190	41.4 (38.4–43.9)	92	20.2 (16.9–22.9)
Geographic area						
Atlantic (North)	190	47.0 (44.1–49.5)	179	42.6 (39.7–45.0)	67	10.4 (8.2–12.1)
Eastern	86	31.4 (28.1–33.9)	106	47.9 (43.1–51.4)	45	20.7 (16.2–24.0)
Central	164	50.3 (46.6–53.2)	131	32.5 (29.2–35.1)	65	17.2 (13.5–20.1)
Pacific (West)	49	19.8 (15.9–22.7)	108	46.8 (43.2–49.6)	73	33.4 (29.2–36.7)
Bogotá^c^	46	45.9 (40.5–49.5)	39	39.1 (33.3–43.1)	15	15.0 (9.2–19.0)
National territories (South)	162	37.8 (34.1–40.6)	174	39.9 (36.4–42.7)	82	22.3 (17.6–26.0)
Urbanicity						
Urban	441	40.7 (38.6–42.7)	481	41.0 (38.9–42.8)	224	18.3 (16.2–20.1)
Rural	256	38.0 (35.2–40.4)	256	42.4 (39.2–45.0)	123	19.6 (16.7–22.0)
Ethnic group						
Indigenous	95	30.5 (24.9–34.2)	96	38.7 (31.7–43.3)	66	30.8 (23.8–35.4)
Black *or* Afro-Colombian	70	41.0 (35.8–44.8)	85	41.7 (37.6–44.7)	43	17.3 (13.0–20.5)
Others	530	40.5 (38.5–42.3)	554	41.4 (39.4–43.2)	234	18.1 (16.1–19.9)

^a^All women analysed by ethnic group were *n* = 1,773, another 8 appertained to “Raizal del archipiélago”, who were not analysed because this group do not have a representative sample

^b^It is not correct to calculate the percentages from the “n” presented in this table; these calculations were taken from weight from the values given to each subject

^c^Coefficient of variation is more than 20 % deficiency prevalence therefore variation should be used with caution

Table 2, shows results from the logistic regression analysis. Once the adjustment was performed (by age, ethnic group, geographic area, socioeconomic levels and urbanicity), the predisposing factors for vitamin B12 deficiency included being part of the indigenous ethnic group OR 2.21 (95 % CI 1.13 to 4.34) and living in the Pacific (West) OR 4.41 (95 % CI 2.81 to 6.93), Central OR 1.78 (95 % CI 1.11 to 2.83) or National territories (South) OR 2.34 (95 % CI 1.45 to 3.79).

**Table 2:** Socio-demographic factors associated with vitamin B12 deficiency in a representative sample of pregnant women in Colombia

Characteristics	Bivariate	Adjusted model^a^	Adjusted model^b^	Adjusted model^c^
Age (years)^d^				
13 to 17	1.02 (0.64–1.62)	1.02 (0.64–1.62)	1.14 (0.68–1.91)	1.14 (0.68–1.91)
30 to 49	1.15 (0.77–1.70)	1.15 (0.77–1.70)	1.14 (0.76–1.70)	1.14 (0.76–1.70)
Socioeconomic levels^e^				
Level I	1.18 (0.68–2.06)	1.18 (0.67–2.08)	1.23 (0.70–2.17)	1.20 (0.67–2.16)
Level III	1.21 (0.56–2.61)	1.19 (0.55–2.58)	1.32 (0.60–2.92)	1.30 (0.59–2.89)
Level IV or more	1.36 (0.76–2.43)	1.35 (0.75–2.41)	1.40 (0.77–2.57)	1.40 (0.76–2.56)
Geographic area^f^				
Eastern	***2.25 (1.39–3.66)***	***2.26 (1.39–3.68)***	***2.21 (1.35–3.61)***	***2.21 (1.36–3.62)***
Central	***1.80 (1.13–2.86)***	***1.79 (1.12–2.85)***	***1.78 (1.12–2.85)***	***1.78 (1.11–2.83)***
Pacific (West)	***4.34 (2.82–6.69)***	***4.34 (2.81–6.68)***	***4.42 (2.82–6.92)***	***4.41 (2.81–6.93)***
Bogotá	1.53 (0.81–2.87)	1.51 (0.80–2.84)	1.44 (0.74–2.78)	1.41 (0.73–2.74)
National territories (South)	***2.49 (1.55–3.98)***	***2.49 (1.55–3.98)***	***2.33 (1.44–3.77)***	***2.34 (1.45–3.79)***
Urbanicity^g^				
Rural	1.09 (0.78–1.52)	1.09 (0.79–1.52)	0.95 (0.66–1.36)	0.95 (0.66–1.36)
Ethnic group^h^				
Indigenous	***2.13 (1.15–3.95)***	***2.12 (1.15–3.93)***	***2.22 (1.13–4.36)***	***2.21 (1.13–4.34)***
Others	1.06 (0.67–1.65)	1.06 (0.68–1.65)	1.35 (0.80–2.29)	1.35 (0.80–2.29)

Odds ratios (95 % confidence interval)

^a^adjusted by age

^b^adjusted by ethnic group, geographic area, socioeconomic levels and urbanicity

^c^adjusted by age, ethnic group, geographic area socioeconomic levels and urbanicity

^d^reference group: 18 to 29

^e^reference group: level II

^f^reference group: Atlantic (North)

^g^reference group: urban

^h^reference group: black or afro-Colombian

Significant odds ratios are shown in bold

## Discussion

Maternal nutritional status is one of the most important indicators of perinatal risk and the birth weight and health of a newborn [12]. We found that approximately 2 out of every 5 pregnant women had a marginal vitamin B12 deficiency (41.3 %, 95 % CI 39.4 % to 43.4 %). In addition, vitamin B12 deficiency was (18.6 %, 95 % CI 16.8 % to 20.3 %) which is a greater prevalence than previously reported (34.5 % in Tukey and 35 % in Canada) [13, 14] in North America and women of childbearing age in Colombia (13.2 %) [10].

Only one previous study had examined vitamin B12 status in non-pregnant Colombian women [15]. Re-analysis of the Colombian National Nutrition Survey data showed that low vitamin B12 (<148 pmol/l) is common in the children (age <18 years) (6.6 % 95 % CI 5.2 % to 8.3 %) and in women of reproductive age (18.5 % 95 % CI 4.4 % to 53.1 %) [15]. Prevalence of marginal vitamin B12 deficiency in the same groups was, 22.5 % (95 % CI 21.1 % to 23.9 %) and 18.8 % (95 % CI 7.7 % to 39.2 %) [15].

The multivariate logistic regression shows that pregnant women belonging to the indigenous ethnic group OR 2.2, (95 % CI 1.1 to 4.3), living in the Pacific region (West) OR 4.4, (95 % CI 2.8 to 6.9), or National territories (South) OR 2.3, (95 % CI 1.4 to 3.7) were associated with a higher probability of serum vitamin B12 deficiency. No variations were observed for ethnic groups, socioeconomic status or urban and rural area. The differences observed in our study are similar to previously reports by Allen [11] which analyzed vitamin B12 deficiency in the Americas. Colombian children living in the Pacific region showed a higher prevalence for vitamin B12 deficiency (30.4 %) while those living in the Central and Caribbean regions showed a lower prevalence (<15 %). Living in the Pacific region was considered a factor associated with vitamin B12 deficiency in pregnant women, similar results published by investigators from the nutrition department of the WHO [16]. Although not measured in this study, frequently in the Pacific region meals are likely to have low phytate levels, because they consist mainly of fast food, eggs and milk [15]. In addition, in Colombia, the Pacific region environments have been associated with aridity, high food prices, limited natural resources, and poor infrastructure development, which often affect the availability of and access to food and health services. Such factors could explain increased malnutrition rates among populations residing in this region. Similar challenges can also be found in the Eastern and South (National territories) areas of the country and may explain the higher prevalence of B12 deficiency found in those regions as compared to more developed areas of the country (Bogota, Central and Atlantic regions) that include large urban centers. Previous studies have also found that pregnant women living in urban areas and in geographic regions with greater economic and structural development in general had higher serum concentrations than those from rural and poorer areas [17–21].

Vitamin B12 is a cofactor of methionine synthase in the synthesis of methionine, the precursor of the universal methyl donor S-Adenosylmethionine (SAMe), involved in different epigenomic regulatory mechanisms and especially in fetal growth [22]. In addition, vitamin B12 plays an essential role in folate metabolism and there is increasing evidence that poor maternal vitamin B12 status may increase the risk of adverse pregnancy outcomes such as neural tube defects (NTD) [23, 24]. Considering the lack of a defined cut-off value for vitamin B12 deficiency in pregnant women, the prevalence of this condition may be overestimated due to a dilution effect in the second trimester of pregnancy. Measurement of the total vitamin B12 concentration in plasma is the usual method for assessing vitamin B12 status [11]. However, neurological and hematological symptoms of depletion can occur in individuals with plasma vitamin B12 concentrations in the low-normal range [25]. Moreover, the plasma vitamin B12 concentration is not a reliable indicator of vitamin B12 status in pregnancy since there is a gradual, physiologically normal decline in the plasma concentration of vitamin B12 during an uncomplicated pregnancy [25]. However, vitamin B12 concentration has been used in at least four nutritional surveys in Latin American countries [1, 17, 26, 27].

This decline is thought to be due to hemodilution, hormonal changes, alterations in the concentration of vitamin B12 binding proteins, or active transport of vitamin B12 across the placenta [28]. Nevertheless, associations between vitamin B 12 marginal deficiency and adverse health outcomes in observational studies are of concern and urgently need further evaluation in clinical trials [29].

On the order hand, several factors can affect vitamin B 12 concentrations, such as individual genetic variation, disease conditions, prescription and other drug use, and pregnancy. Approximately 20 % of pregnant women with lower vitamin B12 concentrations have neither clinical nor metabolic signs of vitamin B 12 deficiency [30]. One of the important causes about lower status of vitamin B12 could be deficient dietary intake [31]. Based on our dietary survey among indigenous in Colombia, the intake of animal protein intake was very low among pregnant women [2, 7]. Herrán et al. [15] reported the high prevalence of vitamin B-12 deficiency in Colombia population may be caused by a low intake of animal source foods (ASF), which is the only dietary source of the vitamin except in the unusual situation where foods are fortified with vitamin B-12 [11]. Thus, increasing ASF intake should reduce the prevalence of vitamin B-12 deficiency among Colombian pregnant women. These results suggest that meat- or dairy-based programs may represent an effective way of improving the vitamin B-12 concentration among antenatal care [32].

Measurement procedure errors and other technological problems produce slightly high or low biomarker concentrations. These factors adversely affect the biomarkers’ sensitivity and specificity and result in false-positive or false-negative classifications of vitamin B 12 deficiency. Systematic bias and spurious results affected some measurement procedures used in the past to derive reference ranges [28]. The cut-off used affects ENSIN-2010 prevalence estimates [11]. Using similar cut-offs to those used in the published literature [8, 14, 33, 34], we found a prevalence of vitamin B 12 deficiency near to 18 % in an representative sample of pregnant women aged ≥18 y with normal renal function.

However, our findings are of particular interest because low vitamin B12 concentrations during pregnancy have been linked to obesity and insulin resistance in adulthood [35, 36]. As previously observed in Colombia [2, 6] and in other countries around the world [8, 14], a higher prevalence of vitamin B12 deficiency was found in the indigenous group or those residing in rural areas (30,8 % and 19,6, respectively). This result is consistent with the results of other studies conducted in developing countries, including India [8] and Turkey [30], Mexico, Central and South America [11], as well as parts of Asia [31]. Vitamin B12 deficiency has been associated with poverty, overcrowding and lack of public services, which can increase the vulnerability of the pregnant population.

In this study, one limitation had to be addressed. Our survey was about the health status of Colombian pregnant women and only such blood indicators as vitamin B12 was measured. Unfortunately, the metabolic markers of vitamin B12, homocysteine (tHcy), methylmalonic acid (MMA) and holotranscobalamin (holoTC), had not been measured. Studies indicated that these markers increased or decreased when the status of vitamin B12 were low [23]. However, tHcy and MMA started to increase at vitamin B12 concentrations above the typical cut-off value (200 pg/mL), and they increased quickly when vitamin B12 decreased from 400 to 200 pg/mL, which suggested that the change of the markers related to the impairment of functioning of the nervous system had already begun before the occurrence of deficiency in vitamin B12 [37]. Nevertheless, the use of serum vitamin B12 as a proxy of vitamin B12 status requires. Further assessment of the metabolic markers of vitamin B12 could be essential among such pregnant women population. Second limitation of the present study was that dietary intake of vitamin B12 was not assessed. Herran et al. [15] determined that the bioavailability of vitamin B12 was greater from dairy products and fish than from meat. In the dietary study we conducted in Sarmiento [2], only 13.4 % of the protein in the diets of the women was derived from eggs and milk, with 40 % of protein coming from meat. Colombia is a country that is geographically, climatically and ethnically diverse. Clearly these differences could affect food supply, dietary practices, and consequently vitamin B12 intakes. Geographical difference was significant between pacific, eastern and central of Colombia, which might result from different dietary patterns and climate. Nevertheless, our findings provided plausible evidence that the deficiency in vitamin B12 was prevalent among Colombian pregnant women. For example, indigenous women were a high-risk population of being deficient vitamin B12. Third, the use of specific cut-off points of serum vitamin B12 to define deficiency and marginal deficiency also deserves comment. We used the conventionally accepted cut-off points of <200 pg/mL (deficiency) and 200 to 300 pg/mL (marginal deficiency) [11]. While these cut-off points may be considered somewhat arbitrary, there is a pathophysiological rationale to their use. MMA and tHcy concentrations associated with anaemia, megaloblastosis and neuropathy decrease substantially when serum vitamin B12 concentrations are greater than <200 pg/mL, even in the absence of clinical manifestations [15]. Because pregnant women may have elevated MMA levels in the presence of vitamin B12 concentrations >200 pgl/mL, a second cut-off point is necessary to capture probable (or marginal) deficiency. Lastly, we could not establish the gestational age at which vitamin B12 concentrations were collected, given that survey asked only "current pregnancy established".

## Conclusion

In general, a significant prevalence of vitamin B12 deficiency was found in the study population and various sociodemographic factors contributed to this problem. It is important to note that many countries in Latin America are at a stage of the nutrition transition similar to Colombia [38–40]. The present study provides reason to suggest that pregnant women attending antenatal clinics in Colombia would benefit from vitamin B12 supplementation. Consequently, lessons learned from the Colombian case through analyses of available surveillance data, as well as from future process and impact evaluation of policies and programs, have the potential to inform malnutrition prevention efforts in other settings [29]. Despite the importance of vitamin B 12 in health is a nutrient that is not routine tested during pregnancy. Therefore, educating the health personnel on the subject is necessary. In addition, coordination with other educational and health and wellness strategies has produced viable and sustainable interventions. This practice would support the Millenium Development Goals [16] to promote the health of mothers and infants in developing countries.
